# Age-Stratified Analysis of the Impact of Hypertension on National Health Insurance Medical Expenditures in Ibaraki, Japan

**DOI:** 10.2188/jea.JE20081027

**Published:** 2010-05-05

**Authors:** Toshimi Sairenchi, Fujiko Irie, Yoko Izumi, Takashi Muto

**Affiliations:** 1 Department of Public Health, Dokkyo Medical University School of Medicine, Mibu, Tochigi, Japan; 2 Department of Health and Welfare, Ibaraki Prefectural Office, Mito, Japan; 3 Environment Health Department, Ministry of the Environment, Tokyo, Japan

**Keywords:** hypertension, medical expenditure, health expenditure, age groups

## Abstract

**Background:**

This retrospective cohort study examined the sex- and age-specific impact of hypertension on medical expenditures.

**Methods:**

In 2006, we analyzed the medical expenditure records of 42 426 Japanese National Health Insurance beneficiaries (16 169 men, 26 257 women) who lived in Ibaraki, Japan, were aged 40 to 69 years, and underwent health checkups in 2002. Blood pressure was classified into 4 categories according to the criteria outlined in the seventh report of the Joint National Committee.

**Results:**

The difference in median total expenditure between the hypertension categories and the normotension category was 119 585 yen (140 360 yen vs 20 775 yen) for men aged 40 to 54 years, 126 160 yen (204 070 yen vs 77 910 yen) for men aged 55 to 69 years, 125 495 yen (158 025 yen vs 32 530 yen) for women aged 40 to 54 years, and 122 370 yen (208 700 yen vs 86 330 yen) for women aged 55 to 69 years. The median total and outpatient medical expenditures markedly differed between patients with stage 1 hypertension and stage 2 hypertension (which included people on antihypertensive medication) in both sexes and all age subgroups. The median total and outpatient medical expenditures were higher among women than among men in all blood pressure categories.

**Conclusions:**

The impact of hypertension on medical expenditures was similar in all age groups. Therefore, from the perspective of medical economics, prevention of the onset of hypertension seems equally important for all age subgroups.

## INTRODUCTION

Hypertension is a powerful risk factor for stroke, coronary disease, cardiac failure, and peripheral artery disease^1^; it is also related to higher health care expenditures.^2^^–^^4^ In Japan, the impact of hypertension on medical care expenditure has been seen among beneficiaries of health insurance plans managed by the government^3^ and the National Health Insurance system.^4^ Several health care programs have been conducted to prevent hypertension.^5^ These prevention programs are expected to decrease total medical expenditures because cardiovascular disease accounts for approximately 22% of total medical expenditure in Japan.^6^

The associations of systolic blood pressure with all-cause mortality and the incidence of and mortality from cardiovascular disease have been reported to be age-dependent among whites,^7^^–^^9^ residents of the entire Asia-Pacific region,^10^ and Japanese,^11^ which suggests that the impact of hypertension on medical expenditure may be age-dependent, in a manner similar to the relationship between hypertension and mortality. However, this remains to be studied.

A better understanding of the effects of age on the relationship between blood pressure and medical expenditures among the Japanese population is extremely important for public health planning and the targeting of prevention programs. Therefore, we examined sex- and age-specific relationships between blood pressure levels and medical expenditures in a large, retrospective, cohort study.

## METHODS

### Data collection

To examine the age-specific association between hypertension and medical expenditures, a large, retrospective, cohort study was carried out. The subjects were selected from Japanese beneficiaries of the National Health Insurance system in Ibaraki, Japan. A total of 46 014 beneficiaries were enrolled. They were aged 40 to 69 years in 2002 (17 763 men and 28 251 women), were beneficiaries from April 2002 through March 2007, and underwent health checkups conducted by the Ibaraki Health Service Association from April 2002 through March 2003. Beneficiaries who were no longer covered by the insurance system, such as those who died or changed their address, were not included. The health checkups were conducted in accordance with local government regulations. The data were collected by the Ibaraki Prefectural Government from local governments and the Ibaraki National Health Insurance Organization, after linkage to medical expenditure records in 2006; all data were depersonalized to ensure anonymity. The protocol of this study was approved by the institutional review board of Dokkyo Medical University School of Medicine.

The data collected included blood pressure measurements, anthropometric measurements, and information from blood samples and from interview questionnaires dealing with smoking status, daily alcohol intake, and history of diabetes. A total of 3588 subjects (1594 men and 1994 women) with incomplete data or a history of heart disease or stroke at baseline were excluded; thus, the study included 42 426 subjects (16 169 men and 26 257 women). The medical expenditure records of the subjects were analyzed in 2006.

### Health checkups

The health checkups were conducted from April 2002 through March 2003. At the health checkups, each subject’s blood pressure in the right arm was measured in the seated position after at least 5 minutes rest by trained observers using standard mercury sphygmomanometers. Height in stockinged feet and weight in light clothing were also measured. The body mass index (BMI) was calculated as weight in kilograms divided by the square of their height in meters.

Non-fasting blood samples were drawn from seated participants into 2 polyethylene terephthalate tubes: one with an accelerator, and the other with sodium fluoride and ethylenediaminetetraacetic acid. Serum total cholesterol and serum triglyceride levels were measured using an enzyme method with an H7350 device (Hitachi, Tokyo, Japan). High-density lipoprotein cholesterol levels were measured in the same laboratory using a direct assay with an H7350 device. The plasma glucose level was measured using the hexokinase/glucose-6-phosphate dehydrogenase method with an H7170 device (Hitachi, Tokyo, Japan). The measurements of serum total cholesterol and high-density lipoprotein cholesterol levels were standardized by the Osaka Medical Center for Health Science and Promotion under the US National Cholesterol Reference Method Laboratory Network (CRMLN). The laboratory in the Osaka Medical Center for Health Science and Promotion has been standardized since 1975 by the CDC-NHLBI Lipid Standardization Program administered by the Centers for Disease Control and Prevention (Atlanta, GA, USA); the laboratory has satisfied the criteria for both precision and accuracy of lipid measurements.^12^ The laboratory has also participated in external standardization and satisfied the criteria for precision and accuracy for the measurement of blood samples by the Japan Medical Association, the Japanese Association of Medical Technologists, and the Japan Society of Health Evaluation and Promotion.

An interview was conducted to determine hypertension medication (yes or no), diabetes mellitus medication (yes or no), smoking status (never smoker, former smoker, current smoker), and alcohol intake (never, sometimes, daily).

### Medical expenditures

The subjects were linked to monthly medical expenditure records with bill dates from May 2006 through April 2007. Most of the records were for visits or hospitalizations in April 2006 through March 2007. The medical expenditure records for pharmacies were included, while the records for dental consultations and treatments were excluded. The medical expenditures were expressed in Japanese yen (100 Japanese yen = 0.94 US dollars or 0.60 euros, according to foreign exchange rates on July 1, 2007).

### Statistical analysis

Using the criteria outlined in the seventh report of the Joint National Committee,^13^ the subjects were classified with regard to their blood pressure into 4 categories: systolic blood pressure <120 mm Hg and diastolic blood pressure <80 mm Hg (normotension), systolic blood pressure 120 to 139 mm Hg or diastolic blood pressure 80 to 89 mm Hg (pre-hypertension), systolic blood pressure 140 to 159 mm Hg or diastolic blood pressure 90 to 99 mm Hg (stage 1 hypertension), and systolic blood pressure ≥160 mm Hg or diastolic blood pressure ≥100 mm Hg (stage 2 hypertension). Subjects on hypertension medication were included in the stage 2 hypertension group. Diabetes mellitus was defined as a fasting plasma glucose level of ≥7.0 mmol/L (126 mg/dL), a non-fasting plasma glucose level of ≥11.1 mmol/L (200 mg/dl), or use of antidiabetic medication.

Adjusted geometric means for the total sample, outpatients, and inpatients by blood pressure category for each sex and age subgroup (ie, 40–54 years and 55–69 years) were calculated using the least-square method. For the participants with 0 yen expenditures, logarithmic transformations were performed by replacing 0 yen with 1 yen. Covariates included age (years), BMI, serum total cholesterol level (mmol/L), high-density lipoprotein cholesterol level (mmol/L), log-transformed triglyceride level (mmol/L), fasting status (yes or no), diabetes mellitus (yes or no), smoking status (never smoker, former smoker, current smoker), and alcohol intake (never, sometimes, daily). An analysis of covariance was conducted to test the association between blood pressure and medical expenditures. The analysis was repeated with the interaction term of (age group) × (blood pressure category).

All statistical analyses were conducted using SAS, version 9.1 (SAS Institute, Inc., Cary, NC, USA).

## RESULTS

The subjects’ baseline characteristics by blood pressure category are shown in Table 1. Age, blood pressure, BMI, total cholesterol level, triglyceride level, diabetes mellitus, smoking status, and drinking habits significantly differed by blood pressure category in men. In addition to these variables, high-density lipoprotein cholesterol level also significantly differed in women.

**Table 1.** Baseline characteristics, by blood pressure category, of 16 169 male and 26 257 female National Health Insurance beneficiaries in Ibaraki, Japan 2002

Sex and variable	Blood pressure category				*P* for difference^b^

	Normal	Pre-	Stage 1	Stage 2^a^	
Men					
No. of participants	3309	5866	2874	4120	
Age, years	56.5 ± 8.4	58.6 ± 7.9	60.1 ± 7.4	62.2 ± 6.2	<0.0001
Blood pressure, mm Hg					
Systolic	109.6 ± 6.8	127.7 ± 6.5	143.5 ± 7.5	144.7 ± 16.3	<0.0001
Diastolic	67.9 ± 6.2	77.4 ± 6.5	85.7 ± 7.6	85.4 ± 11.0	<0.0001
Body mass index, kg/m^2^	22.9 ± 2.7	23.7 ± 2.8	24.3 ± 2.9	24.7 ± 2.9	<0.0001
Total cholesterol, mmol/L	5.3 ± 0.9	5.3 ± 0.9	5.3 ± 0.9	5.3 ± 0.9	0.0168
HDL-cholesterol, mmol/L	1.4 ± 0.4	1.4 ± 0.3	1.4 ± 0.4	1.4 ± 0.4	0.2994
Triglycerides, mmol/L	1.5 ± 1.0	1.8 ± 1.2	1.9 ± 1.4	1.8 ± 1.2	<0.0001
History of diabetes, %	7.1	9.3	10.9	13.6	<0.0001
Smoking status, %					<0.0001
Never	21.2	22.3	21.5	23.5	
Former	27.3	32.3	36.4	39.7	
Current	51.4	45.4	42.1	36.8	
Drinking habit, %					<0.0001
None	31.7	25.0	21.0	19.0	
Sometimes	27.1	22.2	19.6	18.3	
Daily	41.3	52.8	59.4	62.7	

Women					
No. of participants	7480	9371	3307	6099	
Age, years	56.4 ± 7.8	59.4 ± 7.1	60.8 ± 6.5	62.3 ± 5.6	<0.0001
Blood pressure, mm Hg					
Systolic	108.1 ± 7.4	127.9 ± 6.2	144.8 ± 6.4	142.6 ± 16.5	<0.0001
Diastolic	66.0 ± 6.4	75.5 ± 6.7	83.2 ± 7.7	81.8 ± 10.3	<0.0001
Body mass index, kg/m^2^	22.2 ± 2.8	23.2 ± 3.0	23.9 ± 3.2	24.6 ± 3.4	<0.0001
Total cholesterol, mmol/L	5.5 ± 0.9	5.7 ± 0.9	5.8 ± 0.9	5.7 ± 0.8	<0.0001
HDL-cholesterol, mmol/L	1.6 ± 0.4	1.6 ± 0.4	1.6 ± 0.4	1.5 ± 0.4	<0.0001
Triglycerides, mmol/L	1.2 ± 0.7	1.4 ± 0.9	1.5 ± 0.9	1.5 ± 0.9	<0.0001
History of diabetes, %	2.7	4.2	5.5	9.0	<0.0001
Smoking status, %					<0.0001
Never	87.9	92.3	92.6	93.1	
Former	2.3	1.7	1.5	1.7	
Current	9.8	6.0	5.9	5.2	
Drinking habit, %					<0.0001
None	74.0	76.6	77.5	79.8	
Sometimes	20.0	17.9	16.8	14.9	
Daily	6.0	5.5	5.7	5.3	

^a^Includes subjects on antihypertensive medication. ^b^Using analysis of variance for age, systolic blood pressure, diastolic blood pressure, body mass index, total cholesterol, HDL-cholesterol and triglycerides, and the χ^2^ test for history of diabetes, smoking status, and drinking habit.

Abbreviation: HDL, high density lipoprotein.

Sex- and age-specific medical expenditures in 2006 by blood pressure category in 2002 are shown in Table 2. The difference in median total expenditure between the hypertension categories and the normotension category was 119 585 yen (140 360 yen vs 20 775 yen) for men aged 40 to 54 years, 126 160 yen (204 070 yen vs 77 910 yen) for men aged 55 to 69 years, 125 495 yen (158 025 yen vs 32 530 yen) for women aged 40 to 54 years, and 122 370 yen (208 700 yen vs 86 330 yen) for women aged 55 to 69 years. The difference in median outpatient expenditure between the hypertension categories and the normotension category was 118 215 yen (138 520 yen vs 20 305 yen) for men aged 40 to 54 years, 121 510 yen (195 450 yen vs 73 940 yen) for men aged 55 to 69 years, 123 390 yen (155 710 yen vs 32 320 yen) for women aged 40 to 54 years, and 129 250 yen (203 910 yen vs 84 660 yen) for women aged 55 to 69 years. The median total and outpatient medical expenditures were markedly different between patients with stage 1 hypertension and stage 2 hypertension in both sexes and all age subgroups. The median total and outpatient medical expenditures were higher among women than among men in all blood pressure categories.

**Table 2.** Sex- and age-specific medical expenditures in 2006, by blood pressure category in 2002, for 16 169 men and 26 257 women covered by National Health Insurance in Ibaraki, Japan

Sex, age, and blood pressure ​ category in 2002	No. of participants	Median (interquartile range) of medical expenditures in 2006 (Japanese yen)	

		Total	Outpatient
Men			
40–54 y			
Normal	1382	20 775 (0–74 460)	20 305 (0–71 600)
Pre-	1869	20 090 (0–84 820)	19 960 (0–78 460)
Stage 1	691	37 730 (4000–144 500)	37 190 (3500–135 840)
Stage 2^a^	563	140 360 (45 580–225 090)	138 520 (44 960–214 760)

55–69 y			
Normal	1927	77 910 (17 370–206 530)	73 940 (17 090–184 390)
Pre-	3997	82 580 (16 620–209 680)	77 110 (16 140–184 220)
Stage 1	2183	109 690 (19 150–231 240)	102 730 (18 740–208 550)
Stage 2^a^	3557	204 070 (127 660–334 240)	195 450 (124 450–299 800)

Women			
40–54 y			
Normal	2963	32 530 (8440–93 210)	32 320 (8440–88 990)
Pre-	2330	39 880 (8670–115 810)	39 395 (8660–113 150)
Stage 1	606	55 290 (11 100–157 050)	53 400 (11 100–149 780)
Stage 2^a^	642	158 025 (99 030–250 270)	155 710 (96 300–241 770)

55–69 y			
Normal	4517	86 330 (25 390–187 990)	84 660 (25 150–178 760)
Pre-	7041	98 080 (27 660–209 050)	94 820 (27 420–195 470)
Stage 1	2701	115 650 (33 280–229 520)	112 330 (32 450–213 750)
Stage 2^a^	5457	208 700 (135 760–322 640)	203 910 (134 690–301 950)

^a^Includes subjects on antihypertensive medication.

The multivariate (age, BMI, serum total cholesterol level, high-density lipoprotein cholesterol level, log-transformed triglyceride level, fasting status, diabetes status, smoking status, and alcohol intake)-adjusted geometric means for total and outpatient medical expenditures were significantly associated with blood pressure category in both sexes and all age subgroups (data not shown).

## DISCUSSION

The results of this large retrospective cohort study showed that the impact of hypertension on medical expenditure was similar across age groups among beneficiaries of the National Health Insurance system in Japan. The differences in the median total and outpatient medical expenditures between hypertensive and normotensive people were both approximately 100 000 yen. The median total and outpatient medical expenditures were markedly different between subjects with stage 1 hypertension and stage 2 hypertension (which included subjects on antihypertensive medication) among both sexes and all age subgroups. The results show that the use of antihypertensive medication results in high medical expenditure. Therefore, lifestyle modification to avoid both the onset of hypertension and the need for antihypertensive medication can be effective in reducing medical expenditures. In addition, median total and outpatient medical expenditures were higher among women than among men in each blood pressure category. Moreover, to the best of our knowledge, this is the first study to show that hypertension has a similar impact on medical expenditure across age subgroups, even though age has been reported to affect the relationship between hypertension and cardiovascular mortality.^11^

Several studies have reported a positive association between hypertension and medical expenditures.^2^^–^^4^ The Health Promotion Research Committee of the Shiga National Health Insurance Organizations^4^ has shown an association between hypertension and medical expenditures. That study, which involved 4191 National Health Insurance beneficiaries who were aged 40 to 69 years, residents of Shiga, Japan, and followed for 10 years, showed that the crude arithmetic mean total medical expenditure per person per month was 45 947 yen for men with stage 2 hypertension. In contrast, the mean expenditure was 15 009 yen for men with normal blood pressure. The arithmetic mean expenditure was 23 332 yen for women with stage 2 hypertension and 14 222 yen for women with normal blood pressure. The medical expenditures in that study were higher than those in the present study, which may be due to the low medical expenditures of the present study area, as compared with Japan as a whole (http://www.mhlw.go.jp/topics/bukyoku/hoken/iryomap/05/01.html). Kitazawa et al^3^ showed an association between blood pressure and medical expenditures among 2165 Japanese employees with a mean age of 44.8 years who were beneficiaries of government health insurance. The sex- and age-adjusted geometric mean total medical expenditure per person per year was 12 950 yen for subjects with hypertension (systolic blood pressure ≥160 mm Hg or diastolic blood pressure ≥100 mm Hg), while the geometric mean expenditure was 6315 yen for subjects with normal blood pressure (systolic blood pressure <140 mm Hg and diastolic blood pressure <90 mm Hg). The Baltimore Worksite Hypertension Control Project,^2^ a study of 3601 employees of the state of Maryland, also showed a positive association between hypertension and medical expenditure. The results of the present study are consistent with these previous studies with regard to the positive association between hypertension and medical expenditure.

Compared to previous studies,^2^^–^^4^ a strength of the present study is the use of a large sample size in which sex- and age-stratified analysis is possible. In addition, all blood samples were measured using the same device, the same reagent, and the same quality control program.

The present study also had several limitations. The possibility of selection bias cannot be ruled out, and the generalizability of the present study is uncertain because (1) the medical expenditure per person was lower in the present study areas than in Japan as a whole (see http://www.mhlw.go.jp/topics/bukyoku/hoken/iryomap/05/01.html), and (2) the participants were limited to people who underwent health checkups. However, the findings may be applicable to persons who underwent health checkups in other regions. A second limitation of the present study is that there was a short interval between the health checkups and the recording of medical expenditures. Furthermore, beneficiaries who were no longer covered, such as those who had died or changed address, were not included. Thus, medical expenditures, especially for inpatients, may be underestimated. Third, medical expenditures were not classified by disease. Further investigation is therefore warranted to completely elucidate the long-term and disease-specific impact of age on the relationship between hypertension and medical expenditures.

In conclusion, the impact of hypertension on medical expenditures was similar in all age groups. Therefore, from the perspective of medical economics, the prevention of the onset of hypertension seems equally important for all age subgroups.
